# Supplementary material for: Adaptation of CD4 in gorillas and chimpanzees conveyed resistance to simian immunodeficiency viruses
Source: bioRxiv. 2024 Mar 25:2023.11.13.566830. Originally published 2023 Nov 13. Preprint. [Version 2] doi: 10.1101/2023.11.13.566830 (PMC10680607; doi:10.1101/2023.11.13.566830)
Supplement: 2 [file NIHPP2023.11.13.566830v2-supplement-2.pdf]

bioRxiv preprint doi: https://doi.org/10.1101/2023.11.13.566830; this version posted March 25, 2024. The copyright holder for this preprint (which was not certified by peer review) is the author/funder, who has granted bioRxiv a license to display the preprint in perpetuity. It is made available under aCC-BY 4.0 International license.

# Supplemental Figures and Table

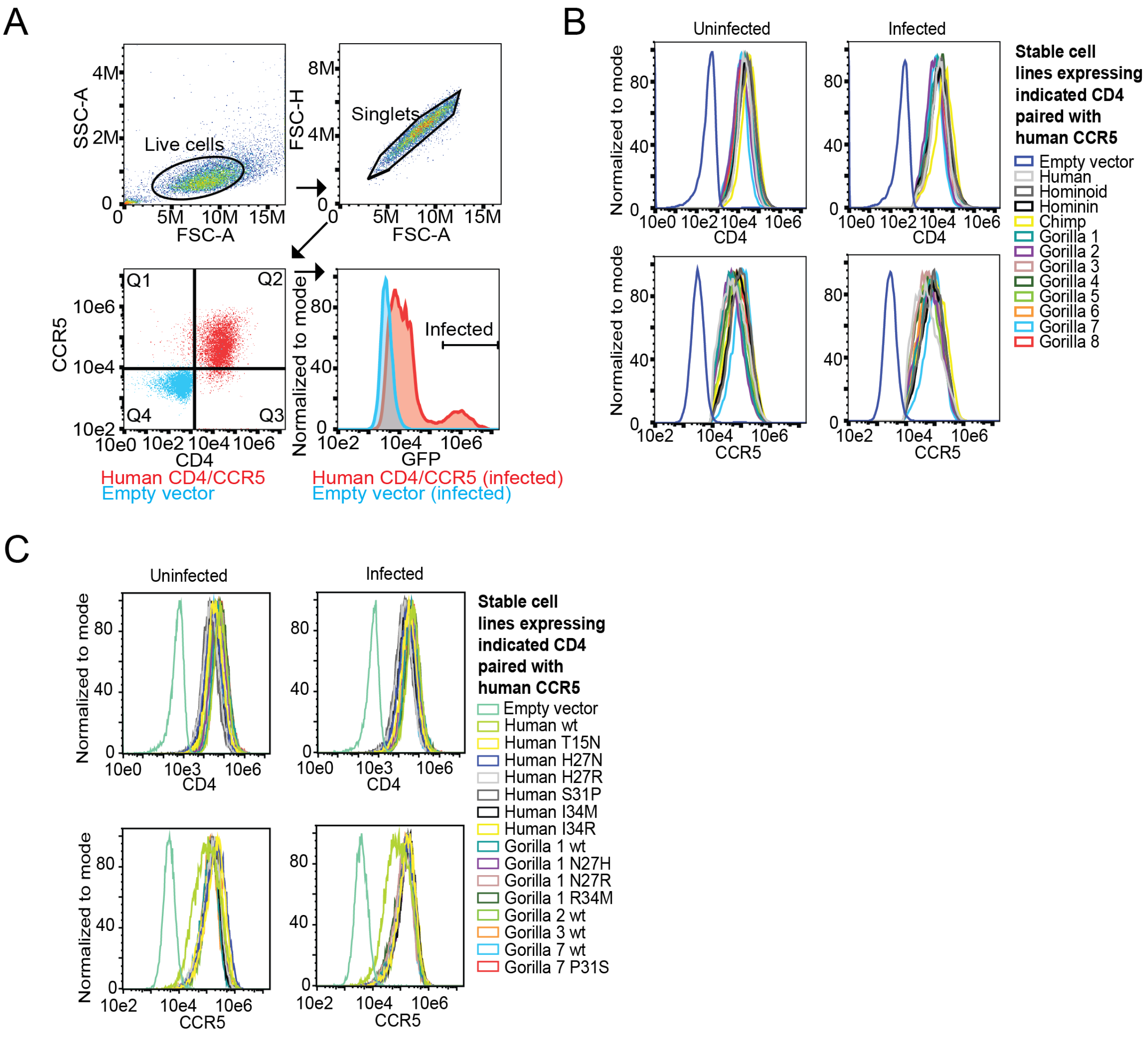


**Figure S1. Flow cytometry gating strategy.** (**A**) Collected events were selected for live cells and singlets based on forward and side scatter values. Singlets were gated for CD4 and CCR5 fluorescent signal and then the double-positive population (Q2) was further analyzed for viral infection based on a shift in GFP fluorescence compared to virus exposed cells lacking CD4/CCR5 receptors (empty vector transduced cells). (**B**) Expression levels for CD4 and CCR5 were compared amongst all stable cell lines under uninfected and infected conditions, demonstrating that viral infection does not impact receptor expression levels. For empty vector control cells, singlets were used for comparison. Data shown are representative of multiple independent experiments.

bioRxiv preprint doi: https://doi.org/10.1101/2023.11.13.566830; this version posted March 25, 2024. The copyright holder for this preprint (which was not certified by peer review) is the author/funder, who has granted bioRxiv a license to display the preprint in perpetuity. It is made available under aCC-BY 4.0 International license.

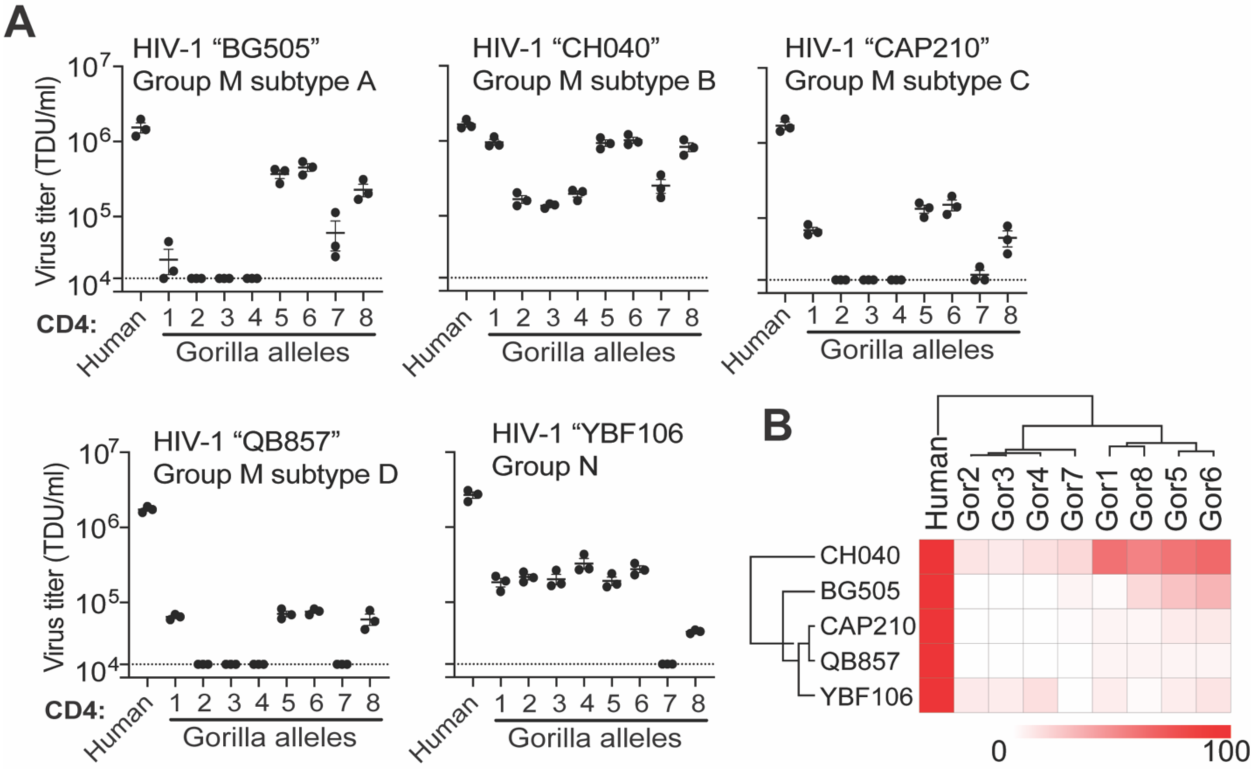


**Figure S2.** **Gorilla CD4 alleles differentially support entry of HIV-1. (A)** HIV-1ΔEnv-GFP viruses were pseudotyped with Envs (top of graphs) from globally diverse HIV strains. Cf2Th cells stably expressing human CCR5 and various CD4s (X-axis) were infected with various volumes of these pseudoviruses and then analyzed by flow cytometry 48 hours post infection. GFP positive cells were enumerated and virus titers (transducing units per milliliter; TDU/mL) were determined for those samples falling within the linear infection range (n = 2 titration points). The mean virus titers obtained from each of three independent experiments were plotted (dots), with error bars representing the standard error of the mean (SEM). **(B)** Data from each pseudotyped Env in "A" were used to calculate virus titer means normalized to human CD4 expressing cells and were plotted as a heat map. CD4 alleles and Envs were hierarchically clustered to depict similarities in phenotype.

bioRxiv preprint doi: https://doi.org/10.1101/2023.11.13.566830; this version posted March 25, 2024. The copyright holder for this preprint (which was not certified by peer review) is the author/funder, who has granted bioRxiv a license to display the preprint in perpetuity. It is made available under aCC-BY 4.0 International license.

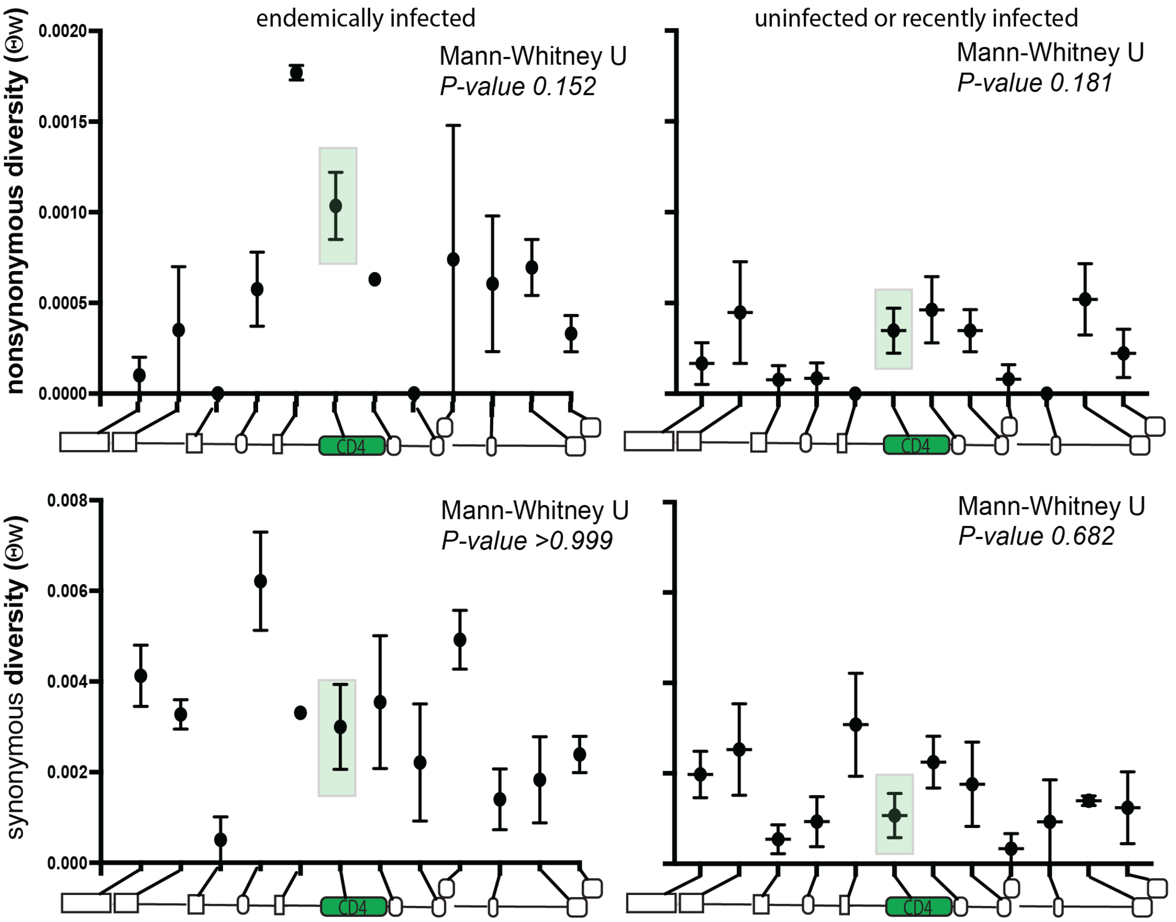


**Figure S3. Single nucleotide polymorphisms in ape species.** Population nucleotide diversity at a locus is estimated either based on the number of single nucleotide polymorphisms (SNPs; Watterson's $\boldsymbol{\theta}_{\omega}$(Watterson, 1975)) or mean pairwise difference between individuals ($\boldsymbol{\theta}_{\pi}$ (Tajima, 1983)). The level of variability based on the number of single nucleotide polymorphisms at a locus ($\boldsymbol{\theta}_{\pi}$) is not significantly different between CD4 and neighboring loci. Y-axis shows the mean and standard error of $\boldsymbol{\theta}_{\pi}$ for synonymous and nonsynonymous nucleotide variants at CD4 and neighboring loci across species endemically infected with immunodeficiency viruses (chimpanzee and gorilla) or recently/uninfected (human, bonobo and orangutans). Schematic along bottom of each graph depicts the relative location of each locus and are as follows 5' to 3': *ZNF384*, *PIANP*, *COPS7A*, *MLF2*, *PTMS*, *CD4*, *GPR162*, *GNB3*, *CDCA3*, *TPI1*, *LRRC23,* and *ENO2*. Mann-Whitney test indicates whether heterozygosity at *CD4* is significantly different than neighboring loci. We observe no difference in the total number of nonsynonymous and synonymous SNPs, represented by $\boldsymbol{\theta}_{\omega}$ between *CD4* and its genomic neighbors in the endemic and un/recently infected species (see also **Table S1**).

bioRxiv preprint doi: https://doi.org/10.1101/2023.11.13.566830; this version posted March 25, 2024. The copyright holder for this preprint (which was not certified by peer review) is the author/funder, who has granted bioRxiv a license to display the preprint in perpetuity. It is made available under aCC-BY 4.0 International license.

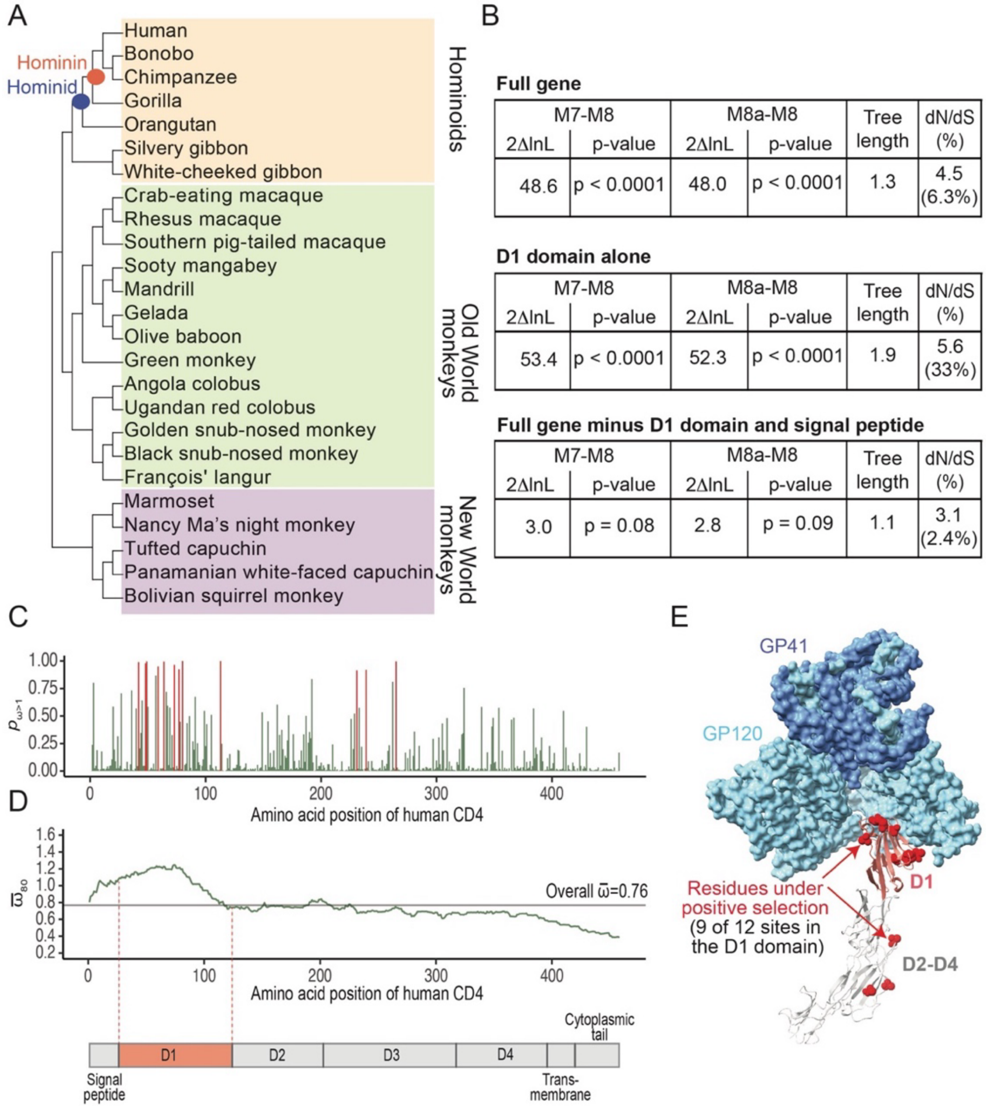


**Full gene**

| M7-M8 | | M8a-M8 | | Tree length | dN/dS (%) |
|---|---|---|---|---|---|
| 2ΔlnL | p-value | 2ΔlnL | p-value | | |
| 48.6 | p < 0.0001 | 48.0 | p < 0.0001 | 1.3 | 4.5 (6.3%) |

**D1 domain alone**

| M7-M8 | | M8a-M8 | | Tree length | dN/dS (%) |
|---|---|---|---|---|---|
| 2ΔlnL | p-value | 2ΔlnL | p-value | | |
| 53.4 | p < 0.0001 | 52.3 | p < 0.0001 | 1.9 | 5.6 (33%) |

**Full gene minus D1 domain and signal peptide**

| M7-M8 | | M8a-M8 | | Tree length | dN/dS (%) |
|---|---|---|---|---|---|
| 2ΔlnL | p-value | 2ΔlnL | p-value | | |
| 3.0 | p = 0.08 | 2.8 | p = 0.09 | 1.1 | 3.1 (2.4%) |

**Figure S4. CD4 is under positive selection in primates.** Previous studies have identified CD4 as an HIV-1 cofactor that is evolving under positive (diversifying) selection (Meyerson et al., 2014; Zhang et al., 2008). However, these studies were limited in that they analyzed *CD4* sequences from a narrow set of primate species (Zhang et al., 2008), or included a larger species panel but lacked the complete *CD4* coding sequence (Meyerson et al., 2014). To extend on these studies, we collected full-length *CD4* sequences from 25 primate species and tested for evidence of site-specific selective pressures using the *codeml* program on the Phylogenetic Analysis by Maximum Likelihood (PAML) package (Yang, 2007a). **(A)** Cladogram of the primate species (n = 25) analyzed in this study. **(B)** The most amino-terminal extracellular domain of CD4 (domain 1, D1) is bound by the primate lentivirus (HIV/SIV) envelope glycoprotein (Env) during entry (Bour et al., 1995). We next sought to assess whether D1 alone is evolving under positive selection (presumably due to selective pressures exerted by SIVs), or if other regions of CD4 are also experiencing selective pressures for diversification. Site-specific selective pressures in primate CD4 (full gene; top), the CD4 D1 domain alone (amino acids 26-123; middle), and CD4 minus the signal peptide and the D1 domain (amino acids 123-458; bottom) were detected using PAML (Yang, 2007a). Positive selection among amino acid sites was tested using two model comparisons, M7 vs. M8 and M8a vs. M8. In each of these comparisons, the null models (M7, M8a) do not allow for sites under positive selection, while the alternative model (M8) does. Tables summarize the likelihood ratio test between the M7-M8 and M8a-M8 models. The 2ΔlnL value (twice the difference in the natural log of the likelihoods) is shown, along with the p-value with which the neutral models (M7 or M8a) are rejected in favor of the model of positive selection (M8). **(C)** To further identify codon sites in CD4 under positive selection, we calculated the posterior probability of ω > 1 (where ω is the dN [nonsynonymous]/dS [synonymous] rate ratio, and values > 1 in the model M8 indicate sites under selection) using the Bayes empirical Bayes approach. Plot of posterior probabilities (ω>1 under maximum likelihood random-sites model M8) for all CD4 sites. Sites under positive selection ($p_{\omega} > 0.9$) are shown in red. (**D**) The posterior mean of ω over a sliding window of 80 amino acids is shown (green line), along with the overall mean of ω across the entire gene (grey line). In both panels C and D, the amino acid positions are shown in relationship to human CD4, and the D1 domain of CD4 is highlighted in orange. **(E)** Cryo-EM structure of an HIV-1 Env trimer in complex with human CD4 (PDB 5U1F) was visualized in ChimeraX (Goddard et al., 2017). Individual gp120 and gp41 subunits are colored in light and dark blue, respectively. The CD4 D1 domain (red) and D2-D4 domains (gray) are shown, with sites under positive selection ($P_{\omega} > 0.9$) shown on the human sequence as red spheres. 9 of the 12 sites passing this stringent cutoff map to the Env-CD4 D1 domain interface.
